# Family caregivers’ perspectives of cultural beliefs and practices towards mental illness in Zambia: an interview-based qualitative study

**DOI:** 10.1038/s41598-022-25985-7

**Published:** 2022-12-10

**Authors:** F. Sichimba, A.‐C. Janlöv, A. Khalaf

**Affiliations:** 1 Department of Psychology, University of Zambia, P.O. Box 32379, Lusaka, Zambia; 2 Faculty of Health Sciences, Kristianstad University, Elmetorpsvägen 15, 291 88 Kristianstad, Sweden

**Keywords:** Psychology, Health care

## Abstract

Many elements of mental illness, including accessibility and utilization of mental health care, are influenced by societal cultural ideas. In Zambia, for example, traditional healers are not recognized, yet a large segment of the population continues to use their services due to their conviction. Despite this, studies on cultural beliefs and practices regarding mental illness in Zambia are scarce. Thus, this study is contextualized in Lusaka, Zambia, with the goal of filling a gap in the knowledge by exploring the cultural beliefs and practices surrounding mental illness as experienced by family caregivers caring for a next-of-kin with mental illness. Using a qualitative exploratory design, a purposeful sample of 15 family caregivers of next-of-kins diagnosed with mental illness were recruited. Data were collected via individual interviews, and analyzed using qualitative thematic analysis. The analysis revealed four main themes: (1) *prevailing beliefs on cause of mental illness*; (2) *encountering social support and neglect*; (3) *recognizing the need of professional help;* and (4) *seeking culturally influenced help*. Findings show that traditional attributions (for example, being bewitched, demon possession and sexual relations with uncleansed widows) are deeply embedded in beliefs and descriptions of what causes mental illness. These beliefs were found to influence not only help-seeking practices but also how people perceived and related to families. Given that beliefs influence caregiver help-seeking, these findings have implications for culturally sensitive practice. The study recommends that public health practitioners consider cultural beliefs and practices when developing health promotion programs, and public health messages.

## Introduction

Culture and its impact on mental health has recently become a topic in Public Health debates. In the last two decades, there has been renewed interest in understanding cultural beliefs on mental illness^1^. However, cultural beliefs and practices around mental illness in Zambia remain largely unknown yet socio-cultural beliefs can contribute to the risk of stigmatization among people living with mental illness and affects mental health service utilization^2^. In Zambia, there are limited mental facilities with only seven general hospitals offering psychiatric services. Coupled with this, the country also has a deficit of trained personnel with literature showing that as of 2014, the country had only three psychiatrists for a population of 13 million^3^. However, despite the limited psychiatric hospitals, mental services such as assessment, rehabilitation, inpatient and outpatient treatment services are available but not utilized the way they should due to reliance on traditional medicine^4^.

Family involvement in patient care is well-documented in literature^5^. In most developing societies, caring for the sick by family members is widespread. For example, in Zambia, family caregivers are traditionally expected to provide care for sick family members^6^. Family caregivers are primary carers for people living with mental disorders and serve as source of emotional, physical (helping to eat, bath, take medication and bedside caring of individual is a norm) and provide much needed financial support associated with mental health treatment^7^. Despite widespread involvement of family in caring for next-of-kin living with mental illness, no studies to our knowledge have explored cultural beliefs and practices (the way they handle and deal with persons with mental illness) among family caregivers in Zambia are limited.

Globally, about one billion people live with mental illness^8^. The changing global landscape has exacerbated the mental health challenge still further. For example, armed conflicts, HIV, poverty, globalization, local changes, and now COVID 19 exacerbate the risk of mental illness. By 2015, the World Health Organization reported that one in every four people is likely to be afflicted with mental illness and this figure was projected to rise by 15% in the year 2020. According to the World Health Organization, one out of every eight persons is impacted by mental disorders^9^. In Africa, the situation remains unclear owing to the lack of statistics on the mental illness burden in most countries. In Zambia, for example, there are no statistics on mental illness; nonetheless, hospital evidence suggests that 3.61 and 1.8 per 10,000 people are serviced by the hospitals' catchment area for acute psychotic episodes and schizophrenia, respectively^10^.

The impact of mental illness in most developing countries is worsened by less comprehensive coverage of the mental health system which often covers a small population^11^. This situation is further compounded by cultural beliefs that limit people from accessing mental health services as the majority prefer to seek help from traditional healers^12^. Literature has shown that, though mental health problems in the traditional African belief systems are attributed to spiritual sources^13,14^, there is a lack of attention to traditional understanding of health and belief systems in most of the studies conducted on mental illness. The Social Ecological Model by Bronfenbrenner^15^, underscores the importance of social environment in understanding illness. According to model, the social environment, namely the microsystem, mesosystem, exosystem, macrosystem and chronosystem, has an influence on human behavior and development. Thus, the Social Ecological Model provokes public health practitioners to look beyond the individual and focus on beliefs informed by culture if mental illness and help-seeking behavior are to be understood. This study uses the ecological model to understand caregiver’s beliefs and practice as influenced by the environment.

Culture is defined as a "set of explicit and implicit guidelines that individuals inherit as members of a particular society, and that tells them how to view the world, experience it emotionally, and behave in it in relation to other people, supernatural forces or gods, and the natural environment”. Culture also provides the "current generation with a way of transmitting these guidelines to the next generation—by the use of symbols, language, art, and rituals"^16^. The coming of European missionaries and colonists in the early nineteenth century brought with it several changes in values, not only in the way of life but also in medical provision. This change meant everything that was Zambian was evil, primitive, and at worst uncivilized^17^. Thus, traditional African understanding of health care was pushed outside conventional medical service provision and discourse. Mental health provision was also not spared from these changes as conventional mental health practice was given prominence much to the exclusion of traditional cultural viewpoint of mental illness^18^. Despite the prevalence of modern mental health services, culture continues to influence how people conceptualize the cause of mental illness in general, which may explain the variation in preferred treatment methods. However, most studies on mental illness tend to focus on professional facts and knowledge while ignoring the belief systems that people inherit from their cultures. Opare-Henaku and Utsey^19^ in their study among the Akan people of Ghana underscored that mental illness is a culturally laden phenomenon and beliefs about aetiology vary across cultures. Similarly, Kirmayer and Swartz^20^ points to the inherent weakness in the medical model as it failed to consider culture, thus advocating the need to understand the social underpinnings and local variability of mental illness.

Studies on cultural beliefs about mental illness in Zambia are scarce. According to Ventevogel et al.^21^, scholars cannot afford to ignore cultural understanding if holistic mental health care is to be provided.

### Aim

The aim of this study was to explore cultural beliefs and practices of family caregivers caring for a next-of-kin with mental illness.

## Methods

### Study design

A qualitative exploratory design was used for this study because of its ability to broaden our understanding of people's beliefs, experiences, and behavior^22^.

### Setting

The research was carried out in Lusaka, Zambia. The estimated total population of the city stands at approximately 1.2 million and is one of the fastest growing populations in Africa^23^. Lusaka hosts the biggest mental hospital in Zambia. The hospital is the country's largest referral mental health facility with a bed capacity of 210. Mental health services provided by hospital include psychiatric treatment, rehabilitation, and general care services to both inpatient and outpatient. Schizophrenia, affective disorders, alcohol-related issues, psychotic episodes, and organic brain abnormalities are among the mental illnesses treated in the hospital^4^. As of 2017, Zambia has only 6 psychiatrists, 3 psychologists and 1.43 mental health nurses per 100,000 people^24^. Just like the rest of Zambia, some barriers to mental health services utilization in Lusaka include dependence on traditional healers, poverty, lack of awareness and culture^4^.

### Participants

To explore beliefs and practices, a total of fifteen (15) participants who were living with and caring for a next-of-kin diagnosed with mental illness were recruited in September, 2021 from Chainama Hills Mental Hospital in Lusaka, the out-patient department’s waiting room. Purposive sampling methodology allowed the researchers to select only caregivers who met the inclusion criteria of being primary caregivers, caring for their loved one living with mental illness, and familiarity with the subject under study. Additionally, the purposive sampling aimed for a recruitment of a more diverse caregiver sample such as gender, age, relationship to the person with mental illness, and type of mental illness in their care. No formal data was collected about the mental illness diagnoses, but according to the family caregivers their next-of-kin suffered from a range of mental illnesses including schizophrenia, bipolar disorder, and substance-related psychosis.

Of the 15 caregivers who participated, nine were male and six were female. In addition, four of the participants were single; ten were married and one divorcee. In terms of education level; three had primary education, five had secondary education, and seven had tertiary education. The median caregiver age was 48 years, with the minimum age of 25 years and maximum age of 56 years. Seven out of the 15 caregivers were taking care of their children (of which five were sons and two were daughters) while four caregivers were taking care of their brothers, three caregivers were taking care of their spouses, and one provided care for a nephew.

### Data collection method

The information sheets were distributed by the principal investigator to the family caregivers accompanying their next-of-kin to the targeted hospital’s outpatient department. Family caregivers who met the inclusion criteria and were considered to be eligible participants were then recruited. Before indicating their interest in taking part in the study, the family caregivers were also provided verbal information and urged to ask the researcher any questions they might have. Participants were given the option to select an appropriate day and time for the interviews when they registered to participate.

Individual interviews were used to collect data for the study. The use of in-depth interviews is a common data collection approach in health services research^25^; it enabled the researchers to explore caregiver’s beliefs and practices. An interview guide (Table 1) was developed in line with the research aim of this study and later piloted to ascertain its usefulness in answering the central research question. Following the pilot research, no changes to the interview guide were made. The guide provided topics within which the interviewer (the Principal Investigator) freely asked questions, probed, and made follow-up questions where clarification, further explanation, or illustrative examples were needed. Open-ended questions allowed participants to freely express their opinions while also allowing the researcher to collect detailed descriptions of beliefs and practices. Examples of questions see interview guide below:

**Table 1:** Interview guide.

1. Can you please tell me about what the term mental illness mean to you?
2. Share with me what happened when your next of kin first showed signs of the illness
Probe:
Kindly share your experiences before and after you found out?
What you thought about the condition and how it came about
What people said about condition?
Treatments that were sought and why?
What made you decide to take your next of kin to the hospital?
3. In your community/neighborhood, tell me how people view mental illness?
Probe:
Kindly share your experiences before and after you found out?
What you thought about the condition and how it came about
What people said about condition?
Treatments that were sought and why?
What made you decide to take your next of kin to the hospital?
4. Please share with me the reactions of neighbors, relatives and other members of the community when they found out that your next of kin had mental illness
5. Give me any suggestions based on what you have been through that you think will help families caring for next of kin living with mental illness?

The interviews were conducted at Chainama Hills Mental Hospital, in a quiet private room, free from distraction. Each interview lasted between 45 and 60 min and was digitally recorded and transcribed by the first author. When no new information was given by the last four participants, the data was assumed to have reached saturation and no new interviews were conducted.

### Data analysis

According to Braun and Clarke^26^, thematic analysis involves identification, analysis, and reporting of patterns (themes) within the data. Because thematic analysis is subjective, a triangulation process was initiated in which all three authors participated separately, in different phases of the analysis process. Identifying meaning units, condensing and coding them, and developing subthemes and, finally, themes were all part of this process^26^. The first step was to thoroughly read the transcripts in order to become acquainted with the data. Thereafter, statements with specific meaning to the research goal (meaning units) were identified and condensed. The condensed units aided in the development of the codes. Following that, the analyzed codes were grouped based on similarities and differences to form subthemes, which were then grouped into themes. All three authors then reviewed the analyzed data to ensure agreement on the final sub-themes and themes. The themes were formed in line with a manifest/descriptive level of analysis (see the Supplemental file for examples of the analysis).

### Ethical approval

The research was carried out in accordance with the Helsinki Declaration's recommendations^27^. Ethical approval and permission for this study were obtained from the University of Zambia, School of Humanities and Social Sciences Ethical committee (number HSSREC:2021-MAY-024). Prior to data collection, all participants were availed information about the study and its purpose both orally and in written, after which consent was obtained. In addition, study participants were informed of the voluntary nature of their participation, right to withdraw from the research without any explanation and were assured that no harm would result on account of their participation^28^. Participants were also assured that the data obtained was for research purposes only, their confidentiality would be secured, and that no personal details were going to be reported^27^.

Because of the sensitive nature of the mental illness, it was envisaged that interviews might evoke psychological stress; thus, the author had a referral system in place for family caregivers that needed counseling a while after. Follow-up with the referral services after the study revealed that no participant used the service. Literature has shown that reflexivity involves critical self-reflections of how the researcher's position, social background, and assumptions impact the research process^29^. In qualitative research, where the researcher has control over the process adopting a reflexive stance was important. Having been born and bred in Zambia, and familiar with prevailing attitudes, it was important to take a reflexive stance. The researcher strived to not influence the study with self-bound values and pre-understanding biases. Thus, to record the researchers’ pre-understanding and reflections, a diary was employed before the data collection process. This diary was not used as a data collection method and thus not analyzed further.

## Results

The thematic analysis yielded themes and sub-themes related to beliefs about and practices around mental illness by family caregivers (note that the term caregivers henceforth means family caregivers). One theme emerged on beliefs about mental illness: namely, Prevailing beliefs about cause of mental illness (with three subthemes)*.* On the practices, three themes were identified: *Encountering support and neglect* (with two subthemes), *Recognizing the need of professional help* (with three subthemes), and *Seeking culturally influenced help* (with three subthemes)*.* An overview of the themes and subthemes is presented in Table 2.

**Table 2:** An overview of the sub-themes and themes regarding experiences of family caregivers’ beliefs and practices when caring for next-of-kin with mental illness diagnosis.

Sub-themes	Themes
Viewing supernatural forces as cause of mental illness	Prevailing beliefs about cause of mental illness
Attributing cause of mental illness to individual’s behavior	
Viewing life circumstances as cause of mental illness	
Receiving support from others	Encountering social support and neglect
Struggling with culturally influenced attitudes	
Trying to manage next of kin’s behavior	Recognizing the need of professional help
Trying to manage own tiredness	
Receiving advice from well-wishers	
Seeking spiritual intervention	Seeking culturally influenced help
Seeking traditional help as culturally advised	
Seeking medical help is a fast and convenient solution	

### Prevailing beliefs about cause of mental illness

Within the theme *Prevailing beliefs about cause of mental illness*, three sub-themes were identified related to caregiver beliefs around mental illness: Viewing supernatural forces as cause of mental illness, Attributing cause of mental illness to individual’s behavior, and Viewing life circumstances as cause of mental illness.

#### Viewing supernatural forces as cause of mental illness

The family caregivers reflected beliefs in supernatural forces beyond the individual as being responsible for mental illness. Supernatural reasons included the disregard for the tradition of sexual cleaning and spiritual possession. Majority of the caregivers alluded to the belief that having sex with uncleansed widow or widower, attracted spell and in some instances even the ghost of a dead spouse in form of mental illness.

**“When one loses a spouse, they must be cleansed by the dead spouse's family. If one is not cleansed or refuses to be cleansed and someone has intercourse with such a widow or widower, they will develop a mental illness.” (Caregiver 14).

Closely linked to supernatural beliefs was spiritual possession. Ten of the fifteen family caregivers expressed that jealousy and enmity from friends, family, or neighbors cause mental illness. Caregivers reported that mental illness spell is a route used by jealous people to thwart ones’ progress or success. Caregivers explained when someone is successful and doing well, or making progress in their work or business, people who are jealous are able to cast a mental illness spell through spiritual possession.

#### Attributing cause of mental illness to individual’s behavior

Caregivers’ narratives mirrored the belief that the afflicted individual is responsible and wholly to be blamed for their mental illness on account of their behavior. Thirteen of the fifteen caregivers explained that bad deeds such as disregard for authority of elders, adultery, substance use, failure to follow procedures when using charms as well as stealing someone’s property caused mental illness*.* For example, *unfaithfulness in marriage was said to attract the wrath of God which manifested in form of mental illness*. Also, lack of special regard for elders was reported to attract a bad omen of mental illnesses by eleven caregivers. Lastly, overuse of substances such as alcohol, drugs, and other substances were irresponsible behaviors mentioned by five caregivers that they believed led to mental illness. One of the caregivers pointed out that: “*drinking beer too much and smoking too much … causes mental illness*” (Caregiver 4)*.*

#### Viewing life circumstances as cause of mental illness

Seven caregivers shared the beliefs that life circumstances can lead to mental illness*.* This culturally influenced belief reflected mental illness as stemming from life situations that were beyond individual control such as heredity, poverty, and life stressors. Caregivers reported that challenges of life such as poverty, losing a loved one, and general stress of life made people to over-think and worry resulting into mental illness. A participant reflected this belief saying: “*Problems of life can make one think too much and this can lead to mental illness*” (Caregiver 11). Additionally, three caregivers described how mental illness runs in some families, thus people who have the misfortune of being born in such families find themselves suffering from mental illness. For example, as one caregiver noted: “*Mental illnesses are generational. It could be that the illness has been in the family and someone is unfortunate to have inherited it*” (Caregiver 1)*.*

### Encountering social support and neglect

Encountering social support and neglect in caregiving yielded from receiving support from others and struggling with culturally influenced negative attitudes.

#### Receiving support from others

More than half of caregivers acknowledged getting help from close friends, family members, and neighbors. Support from others included emotional, material, and financial support to the family. Practices such as being emotionally supported through encouragement, gestures of goodwill, provision of material, and financial support in form of helping caregivers with transport or helping them to buy prescribed drugs and taking care of the next-of-kin were all expressed by the interviewed participants. For example, one caregiver said: “*My neighbors have been helpful. They encourage me, provide advise on how to take care of her and usually help to bring her to the hospital, sometimes with money*” (Caregiver 8).

#### Struggling with culturally influenced negative attitudes

While some caregivers shared supportive tendencies, neglectful tendencies were also reported. Eight caregivers described practices mirroring struggle with culturally influenced negative attitudes targeted towards the family as well as the person with mental illness. For instance, a caregiver described: * “People blame my husband for his illness, claiming that he went to a Satanist to seek riches, but the charms backfired. Others even avoid our home because they believe we are Satanists.”* (Caregiver 6).

Further, caregivers reported blaming practices as common and experienced on a daily basis. They described how their sick next-of-kin suffered mistreatment and were frequently viewed as useless, ineffective, and unproductive. Consequently, society did not perceive mentally ill persons as human beings and treated them as outsiders. Caregivers also indicated that their next-of-kin were subjected to public ridicule, thus caregivers often locked them in the house to shield them. Stigma was described as an everyday experience for their next-of-kin as they were perceived as aggressive and could erupt anytime; thus, the practice was to avoid them. Given these practices, caregivers reported struggling every day to negotiate negative attitudes, practices, and stereotypical beliefs even within their own families and community in a quest to take care of their ill next-of-kin. Having a person with mental illness in their homes changed not only how people related to them but also how they were perceived as a family.

### Recognizing the need of professional help

This theme is contextualized on the practice around decision-making in seeking professional help from the hospital. Three subthemes were identified; *Trying to manage next-of-kin’s behavior*, *Trying to manage own tiredness* as well as *Receiving advice from well-wishers* as being at the center of the caregivers' decision to seek professional help from the hospital.

#### Trying to manage next-of-kin’s behavior

The difficulties in managing next-of-kin’s behavior were experienced as a culturally sensitive issue and were thus a common reason for seeking help. All the caregivers interviewed reported failing to manage and cope with the demands of the illness at home. They reported that the next-of-kin’s disruptive behavior such as undressing in public and aggression was not only culturally unacceptable but draining. For aggressive behavior, six caregivers narrated that the decision to take their next-of-kin to the hospital was when they noted that the person was a danger to themselves and a danger to the caregiver. Thus, the consequent fear and the need for safety motivated family caregivers to take their next-of-kin to the hospital. For example, one caregiver narrated “*He became too aggressive, always beating me; I feared he was going to kill me.*” (Caregiver 1).

#### Trying to manage own tiredness

The caregiver quest to manage their own tiredness was also reported to have contributed to the decision to take the next-of-kin to the hospital. Tiredness, being stressed, exhausted, and inability to cope was widely expressed by caregivers as having informed the decision to take the patient to the hospital among caregivers. As stated by one caregiver:

“*She became too much, we tied her up with a chain, she never wanted to stay at home, running away from home, pouring water on herself, I became tired … that’s how they brought her to the hospital”* (Caregiver 3).

More than half of the caregivers reported that, not knowing the source of mental illness was not only emotionally difficult, but also frustrating to them. They also reported being overwhelmed by the illness and being constantly tired influenced the decision to take the next-of-kin to the hospital.

#### Receiving advice from well-wishers

Receiving advice from well-wishers (church mates, neighbors and friends) was perceived as a culturally common practice and it was also instrumental in the caregiver's decision to take the next-of-kin to the hospital. Nine caregivers reported that advice from well-wishers and medical personnel made them take their next-of-kin to the hospital. Examples regarding advice given included managing the condition, cause of illness and where to seek help.

* “People from church are encouraging and always keep us going by praying for her and the family. They remind us that God never fails and that he heals all illnesses. Their support and advice made us bring her to the hospital.”* (Caregiver 15).

### Seeking culturally influenced help

The theme revealed various practices around culturally influenced help-seeking. Help-seeking practices revolved around taking the next-of-kin for different sources of help informed by caregiver beliefs about cause of the illness. When mental illness was spiritual in nature, spiritual intervention was sought, while mental illness linked to bewitchment and failure to perform cultural rites, help was sought from traditional healers. Mental illness caused by misuse of substances, heredity and life stressors, meant that medical help was sought. The subthemes that emerged here were; Seeking spiritual intervention, Seeking traditional help is culturally advised, and Seeking medical help—a fast and convenient solution.

Interestingly*,* caregiver’s narratives showed that even those who sought medical help or traditional help first also reported using spiritual healing alongside other treatments.

#### Seeking spiritual intervention

Despite caregivers reporting various help-seeking practices, spiritual healing through their religious affiliation was reported by all the fifteen caregivers as the first line of treatment sought. Caregivers reported that their faith in God and assurance that God cures all illnesses motivated them to take their loved ones for prayers. “*People said the condition was spiritual. My family advised me to seek spiritual help by taking the patient for prayers.”* (Caregiver, 14)*.*

#### Seeking traditional help is culturally advised

Caregivers who attributed mental illness to supernatural forces and bewitchment sought traditional help. Caregivers narrated that traditional treatment helped to cure mental illness caused by supernatural forces and bewitchment. Thirteen caregivers who reported that mental illness was caused by a curse, or spell due to individuals’ bad deeds or misfortune sought traditional help. For example, one caregiver narrated that: “*You know our society you cannot run away African medicine. People constantly tell you …look for a witchdoctor because this illness is from people.*” (Caregiver 9).

#### Seeking medical help—a fast and convenient solution

In addition, caregivers who attributed the cause of mental illness to life circumstances such as life stressors and heredity sought medical help first. Medical help was also sought by two caregivers who held beliefs that medical help was faster and more convenient. For example, one caregiver said that: “*I decided to bring him to the hospital knowing that medical personnel can be able to examine him and know what to do faster.”* (Caregiver 3).

## Discussion

This study explores cultural beliefs and practices related to mental illness among family caregivers caring for a next-of-kin living with mental illness. Overall, the study revealed varied beliefs and practices around mental illness rooted in socio-cultural explanations. The beliefs, help-seeking behaviors, stigma, and care load are the main findings discussed here.

The findings of this study provide important insight into caregivers' socio-cultural perspectives on mental illness beliefs and practices. The social ecological model of human development proposed by^15^ emphasizes the interdependence of individual and contextual systems. The results of this study reveal that caregiver beliefs and help-seeking practices on mental illness among family caregivers in Lusaka are entrenched in socio-ecological understanding such as supernatural and spiritual beliefs, in line with the ecological model^15^. The findings support Bronfenbrenner's viewpoint, since caregiver’s conceptualization of cause, and decisions about seeking help mirrored the discourse in the socio-cultural context. Caregivers who held religious beliefs, for example, sought divine intervention through spiritual help, whereas those who linked mental illness to life circumstances sought medical help, and those who believed in witches sought traditional help. Noteworthy, some of the beliefs on the cause of mental illness such as sexual relations with uncleansed spouses and contempt for the authority of elders are culturally specific to Zambia and some countries in Africa. The findings in this study are consistent with previous research that has shown a link between socio-cultural beliefs and help-seeking^2^. Thus, if holistic mental health care is to be offered, practitioners must be sensitive to and familiarize themselves with the cultural beliefs that may limit persons' need for support in the culture in which they work. Furthermore, public health messages that aim at promoting mental health should take into account social-cultural views.

A somewhat surprising finding is that even when the next-of-kin got professional medical treatment at the hospital, family caregivers continued to seek other forms of non-medical treatment such as traditional and spiritual help through prayer. This practice might point to how deeply cultural beliefs are engrained. It could well be that pervasive cultural attributions to mental illness engulfed in beliefs may advertently explain why caregiver prefer spiritual and traditional healing methods as they align with contextual philosophies. As noted by Kajawu et al.^30^ in Africa that have found that people seek African traditional treatment methods as it tends to meet cultural expectations as opposed to biomedical help. This finding highlights the need to incorporate socio-cultural knowledge in mental illness interventions that may help family caregivers in seeking appropriate help early and improve access to modern psychiatric services.

Another finding reveals stigmatizing beliefs and practices around mental illness. Traditional beliefs held informed how communities respond and act towards families taking care of a next-of-kin living with mental illness. Notwithstanding the positive support that the participating caregivers had received, the finding revealed a lack of support, and avoidance of the family as common. Taking care of a next-of-kin living with mental illness was experienced as taxing due to negative responses attributed to beliefs held on mental illness. Beliefs such as mental illness is a punishment of the individual by God and results from vices such as “immorality‟ or stealing made families susceptible to stigmatization. These beliefs and constructions that caregivers are forced to face most likely add to their burden and stress. In the African culture, vices such as adultery, and stealing, are strongly discouraged^31^ as they are considered to attract bad omen such as mental illness to those who engage in the vice. Given this culture, it is not surprising that beliefs about the cause of mental illness reflected personal responsibility as informed by culture and put the blame sorely on the individual for their mental illness. The blame and shame beliefs around mental illness seem to lead to negative stereotyping, and discrimination on those living with mental illness and their families. Thus, in keeping with this finding, stigma directed at caregivers made them live in a constant state of stress, worsening their already worsened mental state due to the stress of taking care of their relative. This finding supports Ayalew et al.^32^ study conducted in Ethiopia, which found that stigma contributed to the caregiver burden which harmed the care provided by the family to the person with mental illness. The findings from this study lend support to the socio-ecological theory given that perceived negative practices around mental illness are influenced by beliefs held, which are contextual. Applying Bronfenbrenner^15^ socio-ecological model on the current study’s findings that negative practices for example stigma operate as a process embedded in socio-cultural beliefs informed by context and influence caregiver practices. Our findings indicate that mental illness-related stigma might be a barrier to mental illness medical treatment and management in a hospital setting and can hinder caregivers from seeking appropriate help. This finding resonates with literature that has reported mental illness-related stigma as a barrier to mental illness treatment^33^. It is therefore imperative to develop culturally appropriate public health education programs on mental illness that target beliefs to curb social rejection and stigma. Further, to reduce social rejection and stigma, sensitization programs must form more effective partnerships with and target communities, churches, and traditional leadership such as chiefs and faith healers^34,35^. Furthermore, incorporating traditional and local healing into Zambian health systems may increase cultural relevance, which may promote early help seeking and help reduce social rejection.

### Methodological considerations

To ensure the trustworthiness of the findings, four dimensions as suggested by Lincoln and Guba^36^ namely credibility, transferability, conformability, and dependability; all of which have been adhered to throughout the research process. To help achieve credibility, i.e. the degree to which the research portrays the genuine meanings of the research participants^36^, the researchers recruited only primary caregivers who were knowledgeable about the condition of their next-of-kin. The credibility of the study can also be considered strengthened by following Braun and Clarke^26^ steps to thematic analysis and outlining the procedure used to develop codes, sub-themes, and themes. The researchers additionally attempted to reduce risks to trustworthiness by being reflective in order to avoid researcher bias and by cross-checking conclusions with other sources of findings. Transferability was considered by providing the study context and describing the recruitment process. To avoid the risk of bias due to pre-understanding, the principle investigator was reflective throughout the process of data collection and analysis. In efforts to ensure the conformability of the study the researchers presented direct quotes from caregivers, thus showing that the findings were supported by the data. Further, outlining the research procedure and clearly documenting findings could be seen as strengthening the study's dependability.

## Conclusions

The study revealed that cultural beliefs such as spiritual possession, witchcraft, and failure to perform traditional rite are at the center of family caregiver's attributions of cause of mental illness. This study also found that cultural beliefs shaped practices around help seeking. Given the finding that stigma is dominate, this study recommends that public health practitioners should take into consideration the cultural beliefs and practices in designing appropriate methods for health promotion programs, public health messages, and medical education. Thus, future research should look at how traditional forms of care can be incorporated into conventional medical practice.

## Supplementary Information

Supplementary Information.

## Data Availability

The datasets generated and/or analysed during the current study are not publicly available due [restrictions by the Research and Ethics Committee at University of Zambia to protect the participants’ privacy] but are available from the Principal Investigator (Francis Sichimba) on reasonable request.

## Abbreviations

CSO: Central Statistics Office
COVID-19: Coronavirus disease 2019
MDAC: Mental Disability Advocacy Centre
WHO: World Health Organization
