# Supplementary material for: Family caregivers’ perspectives of cultural beliefs and practices towards mental illness in Zambia: an interview-based qualitative study
Source: Sci Rep. 2022 Dec 10;12:21388. doi: 10.1038/s41598-022-25985-7 (PMC9736699; doi:10.1038/s41598-022-25985-7)
Supplement: Supplementary file 1 — Supplementary Information. [file 41598_2022_25985_MOESM1_ESM.docx]

Supplementary material

Example of the analysis process.

| Meaning Unit | Condensed meaning | Code | Subtheme | Theme |
| --- | --- | --- | --- | --- |
| Having sex with an uncleansed partner (a person who has not been cleansed after the death of a spouse), be it a man or a woman leads to mental illness. (Interview 14) | Sex with an uncleansed partner can lead to mental illness | Disregard for the tradition of sexual cleansing | Viewing supernatural cause of mental illness | Prevailing Beliefs about cause of mental illness |
| People say that he has a spirit or I don’t know what spirit it is, I don’t know how I can explain it properly, but like he has got a spirit, spirit that controls him in the body. (Interview 1) | The spirit stays in one body and them thus mental illness | Spirit possession |  |  |
| Some people get mental illness because someone who is jealous and envious of them has cast an evil spell on them. This person could be a friend or relative. | Mental illness caused bewitchment through an evil spells due to jealousy and envy |  |  |  |
| Thieving or stealing people’s property. Mental illness is a punishment sent by those who one has stolen from. (Interview 9) | Stealing people’s property attacks a spell in form of mental illness | Bad deeds | Attributing cause of Mental illness to individuals behaviour |  |
| Mental illness is due to effects of charms, maybe they didn’t carry out an instruction accordingly when using charms. People forget the instructions given on how to use those charms and use them in the wrong way, this can cause mental illness (Interview 13) | Effects of charms due to failure to instructions | procedure failure in the use of charms |  |  |
| Failure to respect elders can also cause curses from elders. In our culture one is required to respect elders failure to which they are consequences of bad lucky such as mental illness (Interview 7) | Curses due to failure to respect elders causes mental illness elderly | Lack of respect attracts curses |  |  |
| Mental illnesses are caused by drug abuse. Like if a person overdoses on a drug they could go mad or if they smoke *Chamba* (marijuana) too much. ( Interview 5) | Overdose of drugs, brings mental illness because the brain gets confused. | Substance abuse |  |  |
| Being born in a family where there is mental illness. Because illness has been in the family and someone is unfortunate to have inherited it (Interview 1) | Mental illness runs in some families | Heredity | Viewing life circumstances cause of as mental illness |  |
| Problems of life can make one think too much and this can lead to mental illness Disappointments such as losing a child or spouse. (Interview 11) | Disappointments of life | Life crisis and grief |  |  |
